# Scaling Dementia Care Across a Health System: Integrating Biomarkers, Decision Support, and Collaborative Care Models

**DOI:** 10.1002/alz70857_099982

**Published:** 2025-12-24

**Authors:** Jennifer Woodward, Timothy Ryan Smith, Jill K Morris, Ryan A Townley, Lindsey Gillen, Eric D Vidoni, Dinesh Pal Mudaranthakam, Adam Parks, Amanda Brunette, Michelle Niedens, Jaime Perales‐Puchalt, Tina Lewandowski, Shellie Ellis, Emily Morrow, Janell Jones, Branden Comfort, Shelley Bhattacharya, Rachel Forcino, Taylor Bucy, Jeffrey M. Burns

**Affiliations:** ^1^ University of Kansas Medical Center, Department of Family Medicine, Kansas City, KS, USA; ^2^ University of Kansas Medical Center, Department of Pediatrics, Kansas City, KS, USA; ^3^ University of Kansas Medical Center, Alzheimer's Disease Research Center, Fairway, KS, USA; ^4^ University of Kansas Medical Center, Kansas City, KS, USA; ^5^ University of Kansas Alzheimer's Disease Research Center, Kansas City, KS, USA

## Abstract

**Background:**

Blood‐based biomarkers (e.g., ptau217) and anti‐amyloid therapies have increased the need for earlier, systematic dementia detection in primary care. However, PCPs often lack the tools, training, and time to incorporate these innovations into routine practice. Recognizing this gap, the University of Kansas Health System (UKHS) has implemented a health‐system‐wide model to empower PCPs and streamline dementia care as part of the Davos Alzheimer's Collaborative Healthcare System Preparedness Accurate Diagnosis Program.

**Methods:**

The Brain Health Care Accelerator program at UKHS is a system‐wide model to transform dementia care across primary care clinics in collaboration with specialty memory care. This approach integrates standardized workflows, clinical decision support tools, blood biomarkers, and collaborative care models to enhance early identification and intervention of mild cognitive impairment and dementia while improving PCP diagnostic confidence. Key components include the “Cognitive Assessment Visit” template, blood biomarker testing (ptau217), and specialized referral pathways to memory care sub‐clinics. The “Cognitive Assessment Visit” provides PCPs with a standardized approach to evaluation, incorporating EHR‐based decision support to guide clinical management. Collaborative care models connect PCPs and memory specialists, ensuring the PCP remains central to the patient's care. If memory specialist support is needed, patients can be referred to: (1) E‐Consult Service for rapid specialist electronic consultation, (2) Complex Diagnostic Clinic for comprehensive evaluations, (3) Anti‐Amyloid Treatment Clinic for eligibility and management of monoclonal antibody therapy, (4) Comprehensive Memory Support Clinic for long‐term disease management and caregiver support.

**Results:**

Data on key metrics—time‐to‐diagnosis, time‐to‐referral, treatment initiation, and health service utilization—are systematically collected to evaluate the impact of this scalable, collaborative model. Initial feedback indicates high interest and engagement among PCPs. We also are conducting qualitative assessment of the feasibility and acceptability of the program from health system leadership. The UKHS Family Medicine Department is set to adopt the program in mid‐2025.

**Conclusions:**

This health‐system‐wide model empowers PCPs with clinical decision support, biomarkers, and collaborative care pathways to improve diagnostic accuracy and expand access to timely, high‐quality care. By bridging primary and specialty memory care, this model provides a scalable solution to meet the rising demand for dementia care and address the specialist shortage.

